# Rapid computation and visualization of data from Kano surveys in R

**DOI:** 10.1186/s13104-018-3945-x

**Published:** 2018-11-28

**Authors:** Reynir S. Atlason, Davide Giacalone

**Affiliations:** 1 SDU Life Cycle Engineering, Dept. of Chemical Engineering, Biotechnology and Environmental Technology, University of Southern Denmark, Campusvej 55, 5230 Odense, Denmark; 2 Circular Solutions ehf., Ljósakur 6, 210 Gardabaer, Iceland; 3 SDU Innovation and Design Engineering, Dept. of Technology and Innovation, University of Southern Denmark, Campusvej 55, 5230 Odense, Denmark

**Keywords:** Kano model, Engineering design, User satisfaction, Product development, R

## Abstract

**Objective:**

The Kano model for user satisfaction is a popular survey-based method used by product designers to prioritize the inclusion and implementation of product features based on users’ requirements. Despite its overall simplicity, a current drawback of the Kano approach is that the data analysis and processing of users’ responses is laborsome and rather prone to human error. To address this drawback, this paper provides and presents a complete code to conduct a rapid yet comprehensive computation and visualization of Kano data in R.

**Results:**

A detailed walkthrough of the code is provided, together with a sample dataset to demonstrate its functionality. The code is encapsulated on a simple function that can substantially decrease the time for evaluating Kano results, speeding up its application in the context of product development.

**Electronic supplementary material:**

The online version of this article (10.1186/s13104-018-3945-x) contains supplementary material, which is available to authorized users.

## Introduction

**Fig. 1:**
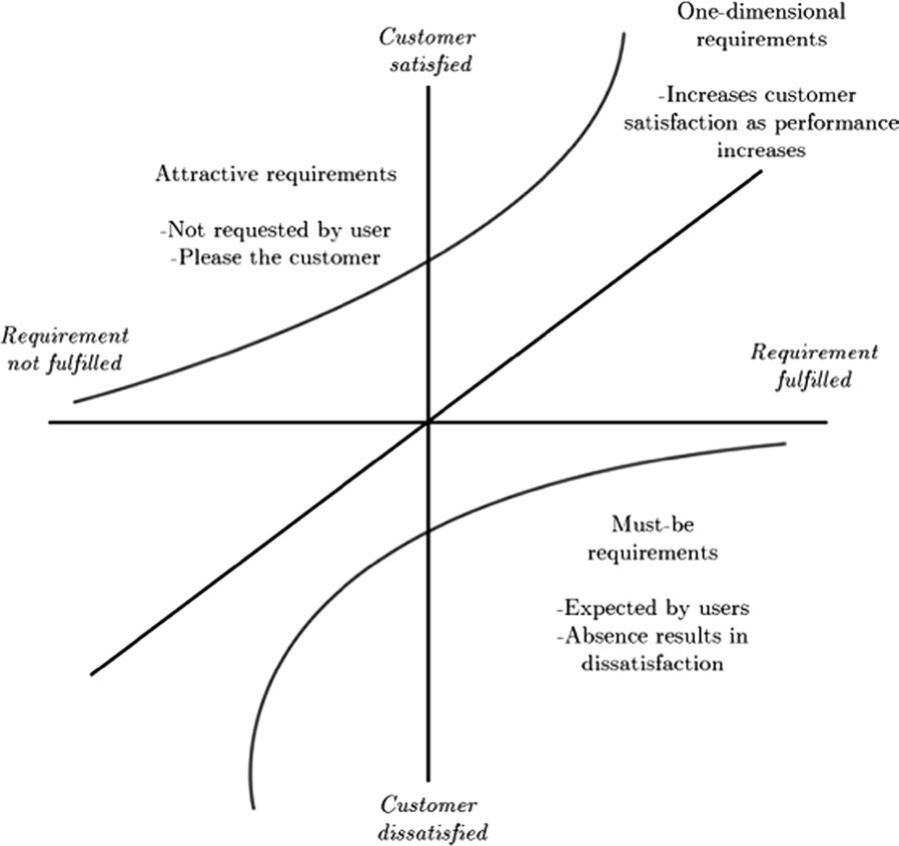
Relationship between implementation of product features and user satisfaction

A Kano survey is a popular tool in product design to inform decisions on whether to implement a particular feature in a product, and to which degree, based on perceived user’ needs, sometimes referred to as “functional requirements” (FRs) [1]. The basic tenet of the Kano approach is that product features affect user satisfaction differently, with some of these relationships being linear while others non-linear (Fig. 1). Depending on the user’ responses, the Kano model classifies FRs in three main classes [2]. The first class is called “Must-be requirements”: features which, when not fulfilled, cause dissatisfaction in the customers. The presence of such requirements is generally taken for granted, but their increasing implementation will, in and of itself, not increase the user satisfaction. For example, mobile phones customers would take for granted the possibility of connecting to the internet, but increasing the speed of the connection beyond a certain point would not impact their satisfaction significantly. The second class is known as “One dimensional requirements”. Such features exhibits a linear relationship with customers’ satisfaction: the more the feature is implemented in the product, the more satisfied the users become. Keeping with the mobile phone example, battery life could be an example of such class. The last class is referred to as “Attractive requirements”, and is often the most sought after by product developers [2], as it includes features not expected by the users (and thus their exclusion from the product would not result in decreased satisfaction), but whose presence may increase the user satisfaction greatly. For example, the possibility of using a mobile phone as a virtual or augmented reality device might fall within that category. Additional FRs classes of the Kano model include “Indifferent” features, whose absence or presence does not affect user satisfaction, “Reverse” FRs, i.e. features whose absence increases user satisfaction, and “Questionable”, when a user indicates that they like both the presence and the absence of a FR. It should be noted that the classification of FR in the Kano framework is subject to change over time (e.g., virtual reality on a phone may be an attractive FR requirement at the time of writing, but in time might become a one-dimensional and eventually a must-be as the technology reaches maturity), and it is also dependent on the context of usage of the products (e.g., seat-back screens on airplanes may be exciting on domestic flights but expected on long distance one), the characteristics of the users, etc.

In product development, the Kano model may help designers solve potential trade-offs by showing which features maximize user satisfaction (see e.g., [3–5], and [6] for application examples). Software solutions for the analysis of these data are, however, very limited. As a result, processing and analyzing this type of data is currently very laborsome and prone to human error. Moreover, the lack of dedicated software solutions may significantly limit its applications in industrial product development. To address this gap, we present a complete R code for the rapid computation and visualization of Kano data, based on the modeling approach proposed by [7].

## Main text

### Quantitative Kano modelling

Though originally a qualitative method [1], quantitative extensions of the Kano model have been proposed in recent years to increase its actionability (e.g. [7] and [8]). A quantitative Kano survey contains questions about the FRs for a target product or service. For each feature, two questions are asked: one functional and one dysfunctional. For example, if being asked about the weight of a mobile phone, the user might be asked “If the phone is as light as a matchbox, how do you feel?”, and then subsequently “If the phone is heavier than a matchbox, how do you feel?”. Each question has five possible outcomes:

1. I like it that way,
2. It must be that way,
3. I am neutral,
4. I can live with it that way,
5. I dislike it that way.

User responses are then collected into a classification table, used to evaluate whether each FR is attractive, one-dimensional, must-be, indifferent, reverse or questionable (Table 1).

**Table 1:** Evaluation matrix for classification

Functional	Dysfunctional				
	1. Like	2. Must be	3. Neutral	4. Live with	5. Dislike
1. Like	Q	A	A	A	O
2. Must be	R	I	I	I	M
3. Neutral	R	I	I	I	M
4. Live with	R	I	I	I	M
5. Dislike	R	R	R	R	Q

A, attractive; O, one dimensional; R, reverse; M, must be; I, indifferent; Q, questionable

After classifying the features, we calculate two values for each of them: user satisfaction (CS) and user dissatisfaction (DS). Those values represent, respectively, the user satisfaction when a FR is fully implemented (CS), and dissatisfaction when a FR is completely excluded (DS). The CS value can be expressed as follows [7]:

1 $$\begin{aligned} CS_i=\frac{f_A+f_O}{f_A+f_O+f_M+f_i} \end{aligned}$$

where $f_A$ denote the number of attractive, $f_O$ the number of one-dimensional, $f_M$ the number of must-be and $f_I$ indifferent responses. Similarly, the following equation can be used to calculate the DS value:

2 $$\begin{aligned} DS_i=\frac{f_O+f_M}{f_A+f_O+f_M+f_i} \end{aligned}$$

Subsequently, two points are located for each FR, which can be plotted as (1, $CS_i$) and (0, $-DS_i$) [7]. Again, these points define, respectively, the user satisfaction when the feature is fully implemented or fully excluded from the product. To find the relationship functions with user satisfaction, one must first identify if the FR is a must-be, one-dimensional or attractive. This is done straightforwardly by considering the mode of the users’ answers for that particular FR. The relationship function can be written as $S=f(x,a,b)$, where *S* is the user satisfaction, *x* the level of fulfilment, *a* and *b* are the adjustment parameters for the Kano categories of user requirements.

For one-dimensional FRs the function is $S=a_{1}x+b_1$ where $a_1$ denotes the slope and $b_1$ is the intercept, denoting the *DS* value when $x=0$. Entering *CS* and *DS* points, as previously calculated, into the equation we get $a_1=CS_i+DS_i$ and $b_1=DS_i$. Therefore, the function for one-dimensional product features can be written as follows [7]:

3 $$\begin{aligned} S_i=(CS_i-DS_i)x_i+DS_i \end{aligned}$$

If the feature is an attractive one, the function is instead considered exponential (Fig. 1), and expressed as $S=a_2e^x+b_2$. We now get $a_2=\frac{CS_i-DS_i}{e-1}$ and $b_2=\frac{-CS_i-DS_i}{e-1}$. We can therefore see that the function for such FRs is [7]:

4 $$\begin{aligned} S_i=\frac{CS_i-DS_i}{e-1}e^{x_1}-\frac{CS_i-eDS_i}{e-1} \end{aligned}$$

For must-be FRs, the function can also be estimated using an exponential function, which in this is $S=a_3(e^x+b_3$). We then acquire $a_3$ and $b_3$ by using $a_3=\frac{e(CS_i-DS_i)}{e-1}$ and $b_3=\frac{eCS_i-DS_i}{e-1}$. The functions for must-be FRs can therefore be plotted as follows [7]:

5 $$\begin{aligned} S_i=-\frac{e(CS_i-DS_i)}{e-1}e^{-x}+\frac{eCS_i-DS_i}{e-1} \end{aligned}$$

### Computation in R

In this section, a R function (called kano) for conducting the analysis explained above is proposed. The kano function does three main things: (1) classification of product features into Kano classes (Table 1), (2) calculation of *CS* and *DS* values, and (3) plotting of relationships functions between individual product features and customers’ satisfaction. To provide a reproducible example, we consider a dataset containing Kano data for six features of a hypothetical product. The present section provides a step-by-step walkthrough of its analysis in R. Both the dataset and the code used for the analysis are provided as Additional files 1 and 2.

Data should be imported as a n*2 dataset. The columns in Additional file 1 dataset consist of the functional and dysfunctional answers, sequentially listed for all FRs and respondents. The answers stored using numerical values from 1 to 5 matching those given in Table 1 (1 = Like, 2 = Must-be, 3 = Neutral, 4 = Live with, 5 = Dislike).

The kano function is of the form function(dataset,FR), meaning that the only input required by the user is to specify the name of the dataset and the number of product features under study. In our example, after importing the data the user can simply run the following code kano(dataset=data,FR=6) (or even simply kano(data,6)) to conduct the analysis. The function prints the output in the console, exports the numerical results to three .csv files, and graphs the functions in the R viewer. Note that the function cannot handle missing data and will return an error if it finds any.

#### Detailed walkthrough

1. After loading some packages needed using library, the first portion of the function runs some diagnostic checks on the dataset. Namely, it checks that the data is of class data.frame, that it does not contain missing values, and that the number of FRs given by the user is correct. If any of these conditions is violated, the function will stop and return a error message explaining the problem to the user.
2. If there are no issues with the data, the function starts by creating two sequences of numbers (*x* and *y*) that will be used for calculating the functions (Eqs. 3–5).
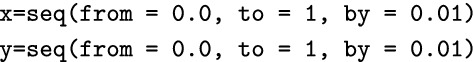

3. It then creates a classification table equivalent to Table 1:
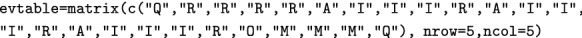

4. The next portion of the code uses the evaluation table to classify each user’s combination of functional and dysfunctional answers, and converts the evaluated answers into a dataframe:
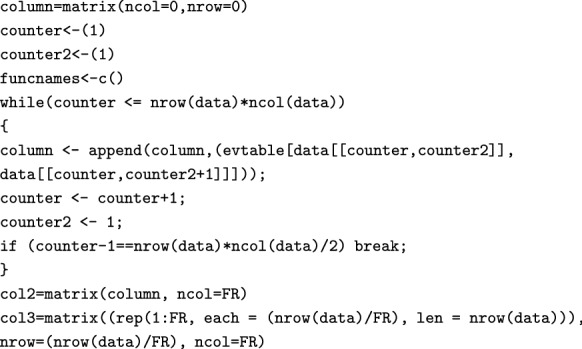

5. Then, the classified data is merged with the original dataset (which now includes the classification of the FRs). We now count the entries for each class and isolate the mode (i.e. the answer with the highest frequency of occurrence) for each FR, and we use this as criterion for classifying each FR into a Kano class:
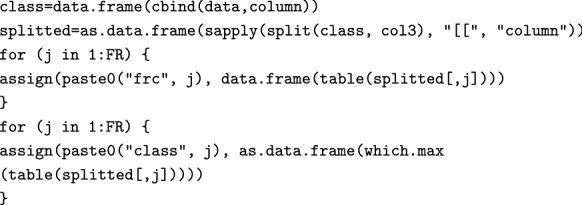

6. From the classified data, CS and DS values associated with each FR are calculated using the following two for loops:
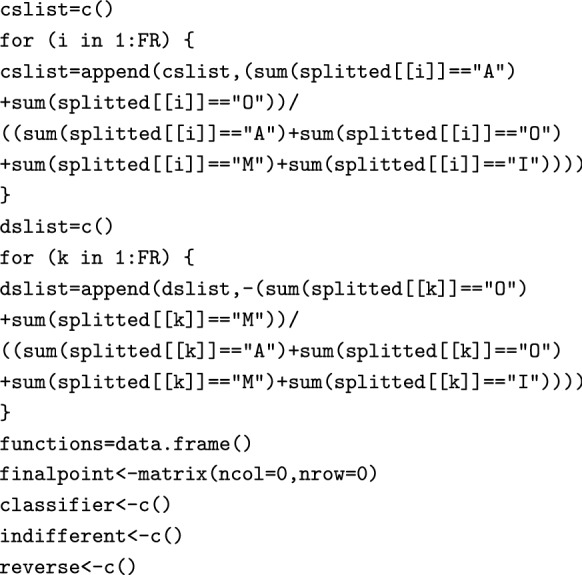

7. We have now finished the classification, and store the results in the splitted data frame. We then move on to calculate the Must-be function (Eq. 5). The will return a function if and only if there actually are any FRs for which the mode is “M”:
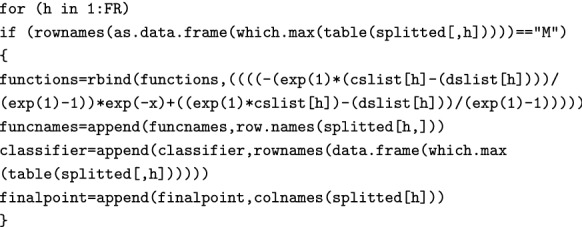

8. We then calculate the function for One-dimensional FRs (Eq. 3). Again, this will happen only if there actually are any FRs for which the mode is ”O”.
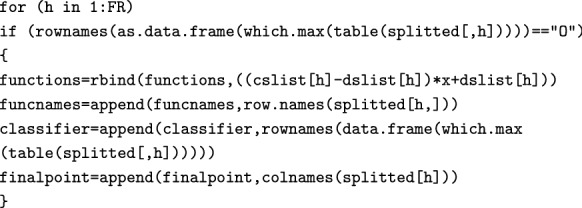

9. Finally, we calculate the function for Attractive FRs (Eq. 4). Again, the code returns only if it finds FRs whose mode is “A”.
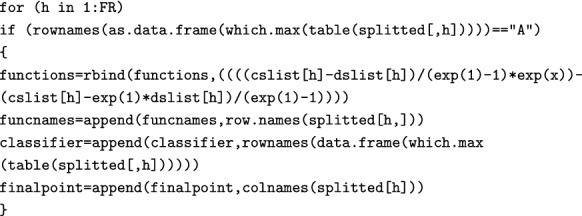

10. Using the following two for loops, we look for Indifferent and Reverse FRs (since these FRs are not typically of interest to product developers, these are only located but not plotted).
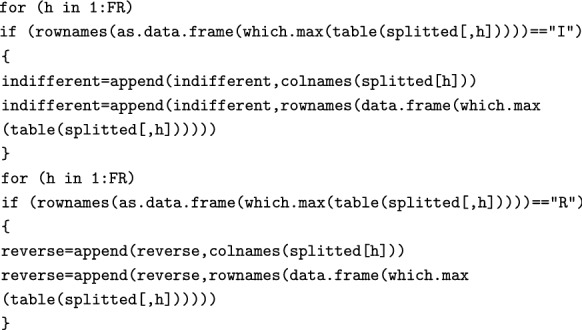

11. As we have located and collected all data points for the One-dimensional, Must-be and Attractive FRs, we can plot them:
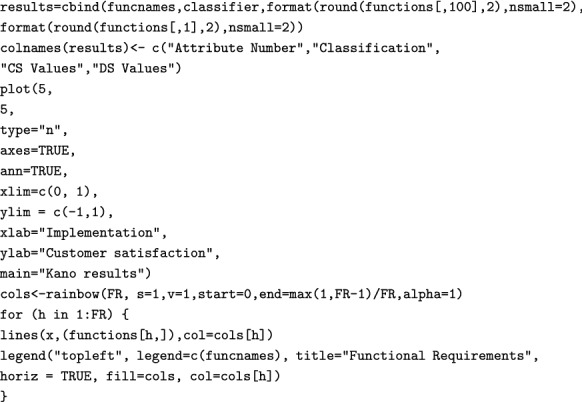

12. The results are written in three different .csv files (one containing A, M and O results, the others containing Indifferent and Reverse FRs), and printed in the R console. R provides first plottable results (for Attractive, Must-be and One-dimensional requirements), then lists Indifferent and Reverse FRs. The results are printed in a data frame where the left column states the number of FRs, the middle column states the classification of the FR, while the two rightern most columns show the values of the functions when $x=1$ and $x=0$ (meaning that the FR is fully implemented or fully excluded, respectively).
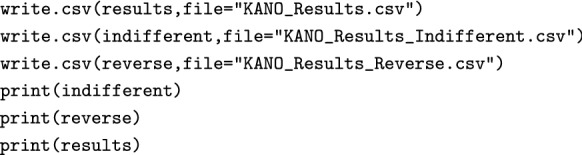

#### Results

In this sample dataset, one FR (“1”) is found to be indifferent, and none to be reverse. Three FRs are Must-be (“2”, “3”, and “5”), one is One-dimensional (“4”), and one is attractive (“6”).

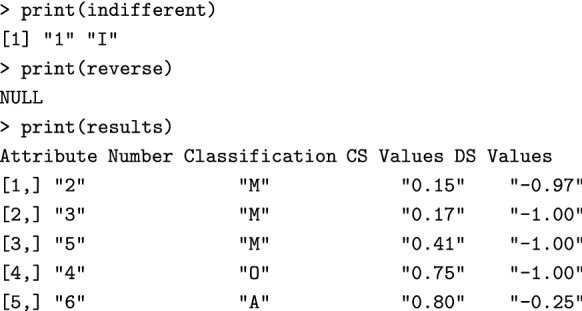



**Fig. 2:**
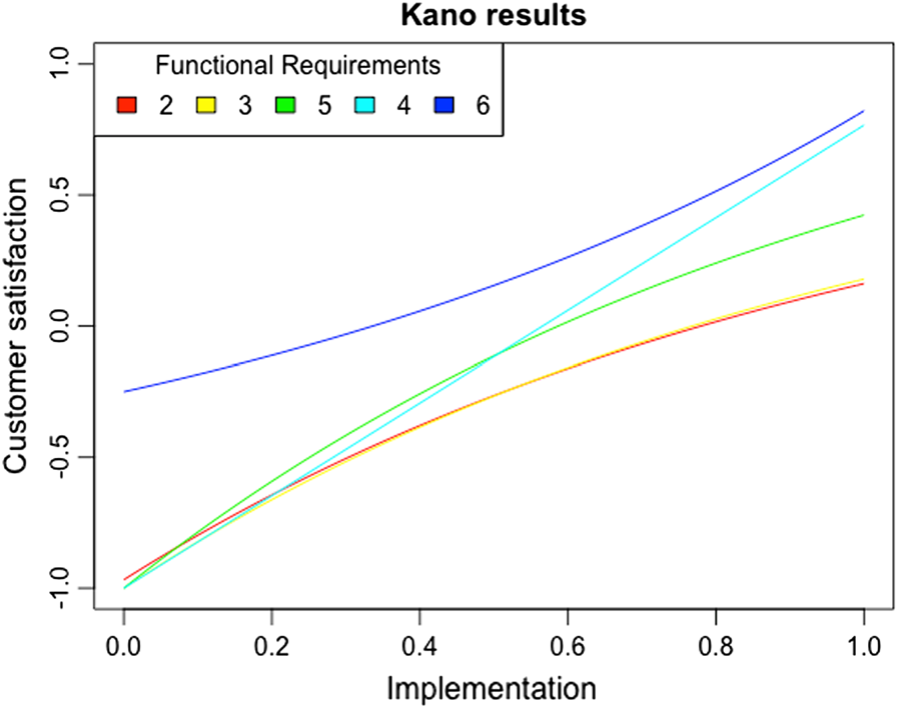
Individual functions relating the degree of implementation of each FR to user satisfaction plotted in the sample dataset

The kano function provides a plot for rapid visualization of the individual functions relating the degree of implementation of each FR to user satisfaction (Fig. 2). Together with the numerical results, such plot can be a useful tool to aid product developers in deciding on which FRs should be prioritized and to which degree they should be implemented. For example, Fig. 2 suggests that four of the original six FRs (“2”, “3”, “4”, and “5”) should definitely be included, as not doing so (corresponding to $x=0$ in Fig. 2) would results in great dissatisfaction for the users. However, with respect to degree of implementation, the one One-dimensional FR (“4”) is clearly the one it makes more sense to maximize, whereas for the three Must-be attributes we can see diminishing returns in terms of user satisfaction with increasing implementation (Fig. 2). Lastly, we have the one Attractive FR (“6”), whose exclusion ($x=0$) would not (much) reduce satisfaction, but its inclusion would result in a marked rise in user satisfaction. Clearly including FR “6”, even to a limited degree, would be a good idea and could also help with differentiating that product from others within the same category.

### Summary

This short paper presented a code for rapid computation and visualization of quantitative Kano data in R, packaged in a simple function (kano(data,FR)) that only requires the user to specify the name of the dataset and the number of FRs to be evaluated. As demonstrated in the worked example, the function allows practitioners to (1) classify FRs according to the Kano framework, (2) compute CS and DS values associated with each FR, (3) compute functions relating each FR to user satisfaction, and (4) plot the results for rapid inspection and visualization. It can assist practitioners and product developers to make informed decisions on which FRs should be implemented (and to which degree) based on Kano results, as well as to make the analysis of this type of data faster and less cumbersome.

## Limitations

This paper only presents a fictional dataset. For examples of real applications of the code in product development context, the reader is referred to two recent papers [3, 4]. Our kano function is based the computational approach proposed by [7], whereas alternatives algorithms for classifying Kano attributes, e.g. [8], are not considered. Finally, the function only considers Kano results at an aggregated level, without possibility for segmentation. The possibility to link results to the user background, as recently proposed in [3], should be a welcome development of the present code.

## Additional file

**Additional file 1.** R code to perform the analysis explained in the paper.

**Additional file 2.** Sample dataset used in this paper.
